# Improved survival in real‐world patients with advanced urothelial carcinoma: A multicenter propensity score‐matched cohort study comparing a period before the introduction of pembrolizumab (2003–2011) and a more recent period (2016–2020)

**DOI:** 10.1111/iju.15014

**Published:** 2022-08-22

**Authors:** Satoru Taguchi, Taketo Kawai, Tohru Nakagawa, Jimpei Miyakawa, Kenjiro Kishitani, Kazuma Sugimoto, Yu Nakamura, Jun Kamei, Daisuke Obinata, Kenya Yamaguchi, Tomoyuki Kaneko, Kanae Yoshida, Sachi Yamamoto, Shigenori Kakutani, Koichiro Kanazawa, Yuriko Sugihara, Mayuko Tokunaga, Akihiko Matsumoto, Yukari Uemura, Yoshiyuki Akiyama, Yuta Yamada, Yusuke Sato, Daisuke Yamada, Yutaka Enomoto, Hiroaki Nishimatsu, Akira Ishikawa, Yoshinori Tanaka, Yasushi Nagase, Tetsuya Fujimura, Hiroshi Fukuhara, Satoru Takahashi, Haruki Kume

**Affiliations:** ^1^ Department of Urology, Graduate School of Medicine The University of Tokyo Tokyo Japan; ^2^ Department of Urology Kyorin University School of Medicine Tokyo Japan; ^3^ Department of Urology Tokyo Teishin Hospital Tokyo Japan; ^4^ Department of Urology Teikyo University School of Medicine Tokyo Japan; ^5^ Department of Urology Musashino Red Cross Hospital Tokyo Japan; ^6^ Department of Urology Jichi Medical University Tochigi Japan; ^7^ Department of Urology Nihon University School of Medicine Tokyo Japan; ^8^ Division of Urology Mitsui Memorial Hospital Tokyo Japan; ^9^ Department of Urology The Fraternity Memorial Hospital Tokyo Japan; ^10^ Department of Urology Tokyo Metropolitan Tama Medical Center Tokyo Japan; ^11^ Biostatistics Section, Department of Data Science, Center of Clinical Sciences National Center for Global Health and Medicine Tokyo Japan

**Keywords:** advanced, bladder cancer, metastatic, pembrolizumab, propensity score matching, urothelial carcinoma

## Abstract

**Objectives:**

Although the treatment strategy for advanced urothelial carcinoma (aUC) has drastically changed since pembrolizumab was introduced in 2017, studies revealing current survival rates in aUC are lacking. This study aimed to assess (1) the improvement in survival among real‐world patients with aUC after the introduction of pembrolizumab and (2) the direct survival‐prolonging effect of pembrolizumab.

**Methods:**

This multicenter retrospective study included 531 patients with aUC undergoing salvage chemotherapy, including 200 patients treated in the pre‐pembrolizumab era (2003–2011; earlier era) and 331 patients treated in a recent 5‐year period (2016–2020; recent era). Using propensity score matching (PSM), cancer‐specific survival (CSS) and overall survival (OS) were compared between the earlier and recent eras, in addition to between the recent era, both with and without pembrolizumab use, and the earlier era.

**Results:**

After PSM, the recent era cohort had significantly longer CSS (21 months) and OS (19 months) than the earlier era cohort (CSS and OS: 12 months). In secondary analyses using PSM, patients treated with pembrolizumab had significantly longer CSS (25 months) and OS (24 months) than those in the earlier era cohort (CSS and OS: 11 months), whereas patients who did not receive pembrolizumab in the recent era had similar outcomes (CSS and OS: 14 months) as the earlier era cohort (CSS and OS: 12 months).

**Conclusions:**

Patients with aUC treated in the recent era exhibited significantly longer survival than those treated before the introduction of pembrolizumab. The improved survival was primarily attributable to the use of pembrolizumab.

**Abbreviations**
aUC: advanced urothelial carcinoma
CSS: cancer‐specific survival
ECOG PS: Eastern Cooperative Oncology Group performance status
MST: median survival time
OS: overall survival
PSM: propensity score matching

## INTRODUCTION

Advanced urothelial carcinoma (aUC), which describes locally advanced or metastatic urothelial carcinoma, has a poor prognosis. Its median survival time (MST) is reported to range approximately 11–15 months following first‐line chemotherapy in both clinical trial^1^, ^2^, ^3^ and real‐world^4^, ^5^, ^6^, ^7^, ^8^, ^9^ settings. For approximately 30 years, there had been no established later‐line regimen for aUC after the failure of first‐line platinum‐based chemotherapy. In 2017, based on the randomized phase III KEYNOTE‐045 trial,^10^, ^11^ pembrolizumab, an immune checkpoint inhibitor, was launched as the first established second‐line regimen, and it has drastically changed the treatment strategy for aUC.^12^, ^13^ According to the long‐term (>2 years) results of the KEYNOTE‐045 trial, patients with platinum‐refractory aUC undergoing pembrolizumab therapy had significantly better survival (MST from the start of second‐line therapy: 10.1 months vs. 7.3 months) and objective response rates (21.1% vs. 11.0%) than those receiving chemotherapy (paclitaxel, docetaxel, or vinflunine).^11^ Although the survival rate in patients with aUC was therefore expected to be improved by the launch of pembrolizumab, studies updating the current survival rates in aUC in the real‐world setting are lacking.^14^ Therefore, the present study assessed the improvement in the survival of real‐world patients with aUC in the pembrolizumab era, as well as the direct survival‐prolonging effect of the drug, using multicenter cohorts with propensity score matching (PSM).

## MATERIALS AND METHODS

### Patients and treatments

This retrospective multicenter study was approved by the Institutional Review Board of the Graduate School of Medicine and Faculty of Medicine, The University of Tokyo (approval number: 10565), as well as that of each participating institution. Because of the retrospective design of the study, the need for written informed consent was waived.

**FIGURE 1:**
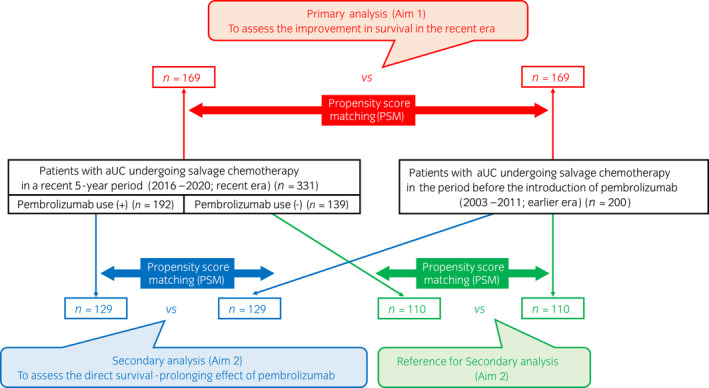
Schema of the study design. In 531 patients with aUC undergoing salvage chemotherapy were included; of these patients, 331 were treated in the recent era (2016–2020), and 200 were treated in the period before pembrolizumab became available (2003–2011; earlier era). In the primary analysis (Aim 1), survival outcomes were compared between the recent era and earlier era cohorts using PSM. In the secondary analysis (Aim 2), which assessed the direct survival‐prolonging effect of pembrolizumab, survival outcomes were similarly compared between patients in the recent era cohort who received pembrolizumab and patients in the earlier era cohort using PSM. As a reference for the secondary analysis (Aim 2), survival was also compared between patients in the recent era cohort who did not receive pembrolizumab and patients in the earlier era cohort after PSM. aUC, advanced urothelial carcinoma; PSM, propensity score matching.

In total, 531 patients from 10 institutions, all of whom underwent salvage chemotherapy for aUC, were included in the study. Of these, 331 patients started first‐line chemotherapy in a recent 5‐year period (from January 2016 to August 2020; recent era), whereas 200 patients were treated in the period before the introduction of pembrolizumab (from April 2003 to July 2011; earlier era; Figure 1). The former cohort was collected from seven institutions, and it included 176 patients treated with pembrolizumab between 2018 and 2020 who were reported in our previous articles.^15^, ^16^ The latter cohort was collected from five institutions, and it was identical to a cohort analyzed in another previous article by our group.^7^ Therefore, we have obtained permission to reuse the previously published material from the publisher (Springer Nature, license number: 5226721033736). Detailed patient demographics according to institutions are presented in Table S1.

For the first‐line salvage chemotherapy, standard platinum‐based regimens, including gemcitabine/cisplatin, gemcitabine/carboplatin, methotrexate/vinblastine/doxorubicin/cisplatin, and dose‐dense methotrexate/vinblastine/doxorubicin/cisplatin, were generally used according to the institutional policy or the physicians' discretion, considering the patient's condition. In the recent era cohort, pembrolizumab was intravenously administered every 3 weeks at a fixed dose of 200 mg for patients with disease progression on or after platinum‐containing chemotherapy given in the neoadjuvant, adjuvant, or salvage setting. All patients underwent evaluations every 1–6 months that included routine blood tests, chest X‐ray, and computed tomography. The patients' charts were comprehensively reviewed, and the status of each patient was assessed through office visits and/or telephone calls.

### Study design, endpoints, and follow‐up

In the primary analysis (Aim 1), which assessed the improvement in survival following the launch of pembrolizumab, cancer‐specific survival (CSS) and overall survival (OS) were compared between the recent and earlier era cohorts using PSM. In the secondary analysis (Aim 2), which assessed the direct survival‐prolonging effect of pembrolizumab, CSS and OS were similarly compared between patients in the recent era cohort who received pembrolizumab and those in the earlier era cohort using PSM. As a reference for the secondary analysis (Aim 2), patients in the recent era cohort who did not receive pembrolizumab were compared with patients treated in the earlier era using PSM (Figure 1). Follow‐up started on the day of first‐line salvage chemotherapy initiation. Follow‐up information was obtained as of May 2014 for the earlier era cohort and as of November 2021 for the recent era cohort.

### Statistical analysis

For PSM, multivariate logistic regression analysis was used to calculate propensity scores, and matching was conducted using the nearest method with a caliper of 0.10 based on the recommendation by our biostatistician (Y.U.). Before and after PSM, the significance of the differences of clinicopathological variables between the recent and earlier era cohorts were evaluated using Student's *t*‐test for continuous variables and the χ^2^ test for categorical variables. Before and after PSM, CSS and OS were estimated using the Kaplan–Meier method and compared using the log‐rank test. The Cox proportional hazard regression model was used for univariate and multivariate analyses for CSS and OS both before and after PSM. All statistical analyses were performed using JMP Pro version 15.0.0 (SAS Institute). *p* < 0.05 was considered significant.

## RESULTS

### Crude data before PSM

Figure S1. presents the overall pembrolizumab use rate in the recent era cohort, which gradually increased over time. Of the 331 patients in the recent era cohort, 192 (58.0%) patients eventually received pembrolizumab during the study period, including first‐line treatment following disease progression on or after neoadjuvant/adjuvant platinum‐containing chemotherapy in 53 patients and later‐line therapy in 139 patients. No patient in the earlier era cohort received pembrolizumab during the study period.

The left half of Table 1 presents the characteristics of all patients (*n* = 531) before PSM. Patients in the recent era cohort were significantly older and had a significantly shorter duration of follow‐up than those in the earlier era cohort, whereas other variables, excluding the first‐line regimens and overall pembrolizumab use, did not differ between the groups.

**TABLE 1:** Characteristics of patients included in the primary analysis (Aim 1) before and after PSM

Parameter	Before PSM				After PSM			
	Total (*n* = 531)	2016–2020 (*n* = 331)	2003–2011 (*n* = 200)	*p*‐value	Total (*n* = 338)	2016–2020 (*n* = 169)	2003–2011 (*n* = 169)	*p*‐value
Age, years, median (IQR)	71 (65–76)	73 (67–77)	68 (62–74)	<0.0001^a^ ^,^ ^b^	71 (64–75)	72 (65–76)	70 (64–75)	0.44^b^
Sex, no. (%)				0.42^c^				0.27^c^
Male	415 (78.2)	255 (77.0)	160 (80.0)		274 (81.1)	141 (83.4)	133 (78.7)	
Female	116 (21.8)	76 (23.0)	40 (20.0)		64 (18.9)	28 (16.6)	36 (21.3)	
ECOG PS, no. (%)				0.17^c^				0.69^c^
≤1	479 (90.2)	294 (88.8)	185 (92.5)		310 (91.7)	154 (91.1)	156 (92.3)	
≥2	52 (9.8)	37 (11.2)	15 (7.5)		28 (8.3)	15 (8.9)	13 (7.7)	
Primary site, no. (%)				0.79^c^				0.95^c^
Bladder	257 (48.4)	160 (48.3)	97 (48.5)		159 (47.0)	78 (46.2)	81 (47.9)	
Upper urinary tract	212 (39.9)	130 (39.3)	82 (41.0)		142 (42.0)	72 (42.6)	70 (41.4)	
Both	62 (11.7)	41 (12.4)	21 (10.5)		37 (11.0)	19 (11.2)	18 (10.7)	
Resection of primary site, no. (%)	341 (64.2)	204 (61.6)	137 (68.5)	0.11^c^	221 (65.4)	108 (63.9)	113 (66.9)	0.57^c^
Prior neoadjuvant/adjuvant chemotherapy, no. (%)	158 (29.8)	106 (32.0)	52 (26.0)	0.14^c^	90 (26.6)	44 (26.0)	46 (27.2)	0.81^c^
Lymph node metastasis, no. (%)	348 (65.5)	211 (63.8)	137 (68.5)	0.26^c^	225 (66.6)	113 (66.9)	112 (66.3)	0.91^c^
Visceral metastasis, no. (%)	290 (54.6)	186 (56.2)	104 (52.0)	0.35^c^	179 (53.0)	89 (52.7)	90 (53.3)	0.91^c^
Lung metastasis, no. (%)	175 (33.0)	104 (31.4)	71 (35.5)	0.33^c^	120 (35.5)	62 (36.7)	58 (34.3)	0.65^c^
Bone metastasis, no. (%)	81 (15.3)	50 (15.1)	31 (15.5)	0.90^c^	53 (15.7)	28 (16.6)	25 (14.8)	0.65^c^
Liver metastasis, no. (%)	58 (10.9)	35 (10.6)	23 (11.5)	0.74^c^	40 (11.8)	21 (12.4)	19 (11.2)	0.74^c^
First‐line regimens, no. (%)				<0.0001^a^ ^,^ ^c^				<0.0001^a^ ^,^ ^c^
GC	237 (44.6)	145 (43.8)	92 (46.0)		150 (44.4)	77 (45.6)	73 (43.2)	
GCa	95 (17.9)	95 (28.7)	0 (0)		47 (13.9)	47 (27.8)	0 (0)	
MVAC	77 (14.5)	5 (1.5)	72 (36.0)		67 (19.8)	4 (2.4)	63 (37.3)	
ddMVAC	10 (1.9)	10 (3.0)	0 (0)		6 (1.8)	6 (3.6)	0 (0)	
Pembrolizumab	53 (10.0)	53 (16.0)	0 (0)		28 (8.3)	28 (16.6)	0 (0)	
Others	59 (11.1)	23 (7.0)	36 (18.0)		40 (11.8)	7 (4.1)	33 (19.5)	
Overall pembrolizumab use, no. (%)	192 (36.2)	192 (58.0)	0 (0)	<0.0001^a^ ^,^ ^c^	93 (27.5)	93 (55.0)	0 (0)	<0.0001^a^ ^,^ ^c^
Follow‐up duration, months, median (IQR)	12 (6–24)	12 (5–23)	12 (7–25)	0.0002^a^ ^,^ ^b^	12 (6–23)	13 (6–23)	12 (7–24)	0.064^b^

Abbreviations: ddMVAC, dose‐dense methotrexate/vinblastine/doxorubicin/cisplatin; ECOG PS, Eastern Cooperative Oncology Group performance status; GC, gemcitabine/cisplatin; GCa, gemcitabine/carboplatin; IQR, interquartile range; MVAC, methotrexate/vinblastine/doxorubicin/cisplatin; PSM, propensity score matching.

^a^ Statistically significant.

^b^ Student's *t*‐test.

^c^ χ^2^ test.

Figure S2 presents the Kaplan–Meier curves before PSM. In total, there were 171 and 170 cancer‐specific deaths and 185 and 171 overall deaths in the recent and earlier era cohorts, respectively. Patients in the recent era cohort had significantly longer CSS (20 months) and OS (18 months) than those in the earlier era cohort (CSS and OS: 12 months). On multivariate analysis before PSM, age (continuous variable), Eastern Cooperative Oncology Group performance status (ECOG PS, ≤1 vs. ≥2), resection of the primary site (no vs. yes), liver metastasis (no vs. yes), and era (2003–2011 vs. 2016–2020) were identified as independent prognostic factors for both CSS and OS (Table 2).

**TABLE 2:** Univariate and multivariate Cox proportional hazard regression analyses of CSS and OS in the primary analysis (Aim 1; *n* = 338)

Parameter	Cutoff	CSS univariate		CSS multivariate		OS univariate		OS multivariate	
		HR (95% CI)	*p*	HR (95% CI)	*p*	HR (95% CI)	*p*	HR (95% CI)	*p*
Age (years)	Continuous	1.02 (1.00 to 1.03) per score	0.026^a^	1.02 (1.00 to 1.04) per score	0.020^a^	1.02 (1.00 to 1.04) per score	0.012^a^	1.02 (1.00 to 1.04) per score	0.0095^a^
Sex	Male	Reference	0.93			Reference	0.80		
	Female	0.98 (0.70 to 1.38)				0.96 (0.68 to 1.34)			
ECOG PS	≤1	Reference	<0.0001^a^	Reference	<0.0001^a^	Reference	<0.0001^a^	Reference	<0.0001^a^
	≥2	4.60 (2.97 to 7.12)		3.31 (2.01 to 5.46)		4.46 (2.89 to 6.90)		3.28 (1.99 to 5.39)	
Primary site	Bladder	Reference	0.89			Reference	0.80		
	Upper urinary tract	0.95 (0.72 to 1.25)				0.93 (0.71 to 1.22)			
	Both	1.04 (0.68 to 1.61)				1.05 (0.68 to 1.60)			
Resection of primary site	No	Reference	0.014^a^	Reference	0.077	Reference	0.0088^a^	Reference	0.050
	Yes	0.71 (0.54 to 0.93)		0.78 (0.59 to 1.03)		0.70 (0.53 to 0.91)		0.76 (0.58 to 1.00)	
Prior neoadjuvant/adjuvant chemotherapy	No	Reference	0.97			Reference	0.83		
	Yes	1.01 (0.75 to 1.34)				0.97 (0.73 to 1.29)			
Lymph node metastasis	No	Reference	0.45			Reference	0.31		
	Yes	1.11 (0.84 to 1.47)				1.16 (0.88 to 1.53)			
Lung metastasis	No	Reference	0.17			Reference	0.17		
	Yes	1.21 (0.92 to 1.58)				1.21 (0.92 to 1.57)			
Bone metastasis	No	Reference	0.0038^a^	Reference	0.39	Reference	0.0065^a^	Reference	0.50
	Yes	1.64 (1.17 to 2.28)		1.18 (0.81 to 1.70)		1.59 (1.14 to 2.21)		1.14 (0.79 to 1.64)	
Liver metastasis	No	Reference	<0.0001^a^	Reference	0.0006^a^	Reference	<0.0001^a^	Reference	0.0005^a^
	Yes	2.53 (1.76 to 3.64)		2.01 (1.35 to 3.00)		2.54 (1.77 to 3.63)		2.02 (1.36 to 2.99)	
First‐line regimens	GC	Reference	0.77			Reference	0.78		
	GCa	0.91 (0.59 to 1.41)				0.87 (0.56 to 1.34)			
	MVAC	1.07 (0.77 to 1.50)				1.04 (0.75 to 1.46)			
	ddMVAC	0.76 (0.24 to 2.40)				0.72 (0.23 to 2.26)			
	Pembrolizumab	0.93 (0.53 to 1.63)				0.88 (0.50 to 1.54)			
	Others	1.28 (0.87 to 1.89)				1.22 (0.83 to 1.80)			
Era	2003–2011	Reference	0.0004^a^	Reference	0.0047^a^	Reference	0.0011^a^	Reference	0.011^a^
	2016–2020	0.61 (0.47 to 0.80)		0.57 (0.39 to 0.84)		0.64 (0.49 to 0.84)		0.62 (0.43 to 0.89)	
Overall pembrolizumab use	No	Reference	0.027^a^	Reference	0.95	Reference	0.031^a^	Reference	0.83
	Yes	0.70 (0.51 to 0.96)		0.99 (0.63 to 1.55)		0.71 (0.52 to 0.97)		0.95 (0.61 to 1.48)	

Abbreviations: CI, confidence interval; CSS, cancer‐specific survival; ddMVAC, dose‐dense methotrexate/vinblastine/doxorubicin/cisplatin; ECOG PS, Eastern Cooperative Oncology Group performance status; GC, gemcitabine/cisplatin; GCa, gemcitabine/carboplatin; HR, hazard ratio; IQR, interquartile range; MVAC, methotrexate/vinblastine/doxorubicin/cisplatin; OS, overall survival.

^a^ Statistically significant.

### Primary analysis (Aim 1): Improvement in survival in the recent era

Table 1 presents the characteristics of patients included in the primary analysis (Aim 1) before and after PSM. For PSM, all pretreatment variables were matched, other than the first‐line regimens and overall pembrolizumab use because these parameters fundamentally differed between the eras. Accordingly, the matched pretreatment variables were age, sex, ECOG PS, primary site, resection of the primary site, prior neoadjuvant/adjuvant chemotherapy, lymph node metastasis, visceral metastasis, lung metastasis, bone metastasis, and liver metastasis. The right half of Table 1 presents the characteristics of 338 patients after PSM. Excluding the first‐line regimens and overall pembrolizumab use, no variables significantly differed between the groups, including the follow‐up duration, which was not matched between the cohorts.

**FIGURE 2:**
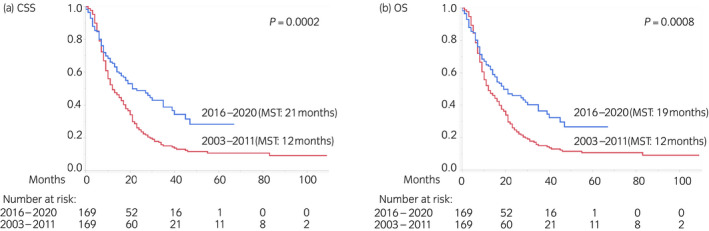
Kaplan–Meier curves after PSM depicting (a) CSS and (b) OS according to the era (2016–2020 vs. 2003–2011) in the primary analysis (Aim 1). CSS, cancer‐specific survival; MST, median survival time; OS, overall survival; PSM, propensity score matching.

Figure 2 presents the Kaplan–Meier curves of CSS and OS in the earlier and recent eras after PSM. In total, there were 82 and 146 cancer‐specific deaths and 87 and 147 overall deaths in the recent and earlier era cohorts, respectively. The recent era cohort had significantly longer CSS (21 months) and OS (19 months) than the earlier era cohort (CSS and OS: 12 months). On multivariate analysis after PSM, age (continuous variable), ECOG PS (≤1 vs. ≥2), resection of the primary site (no vs. yes), liver metastasis (no vs. yes), and era (2003–2011 vs. 2016–2020) were identified as independent prognostic factors for both CSS and OS (Table 2).

### Secondary analysis (Aim 2): Direct survival‐prolonging effect of pembrolizumab

We further performed secondary analyses of patients in the recent era cohort who did or did not receive pembrolizumab versus those in the earlier era cohort to estimate the direct survival‐prolonging effect of pembrolizumab.

**FIGURE 3:**
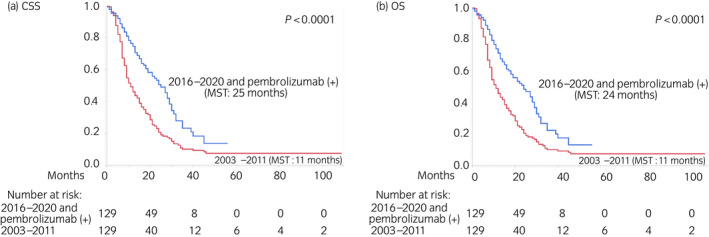
Kaplan–Meier curves after PSM depicting (a) CSS and (b) OS according to the era and overall pembrolizumab use (2016–2020 with pembrolizumab use vs. 2003–2011) in the secondary analysis (Aim 2). CSS, cancer‐specific survival; MST, median survival time; OS, overall survival; PSM, propensity score matching.

Table S3 presents the characteristics of patients included in the secondary analysis (Aim 2) before and after PSM. PSM was conducted similarly to its application in the primary analysis, and 258 patients were selected. Aside from the first‐line regimens and overall pembrolizumab use, both of which were not matched between the groups, no variables differed between the groups, including the follow‐up duration, which was not matched between the cohorts. Figure 3 presents the Kaplan–Meier curves after PSM, depicting CSS and OS according to the era and overall pembrolizumab use (2016–2020 with pembrolizumab use vs. 2003–2011). In total, there were 69 and 117 cancer‐specific deaths and 73 and 118 overall deaths in the recent and earlier era cohorts, respectively. Patients in the recent era cohort who received pembrolizumab had significantly longer CSS (25 months) and OS (24 months) than those in the earlier era cohort (CSS and OS: 11 months). On multivariate analyses after PSM, ECOG PS (≤1 vs. ≥2), liver metastasis (no vs. yes), and era/overall pembrolizumab use (2003–2011 vs. 2016–2020/no vs. yes) were identified as independent prognostic factors for both CSS and OS (Table S4).

**FIGURE 4:**
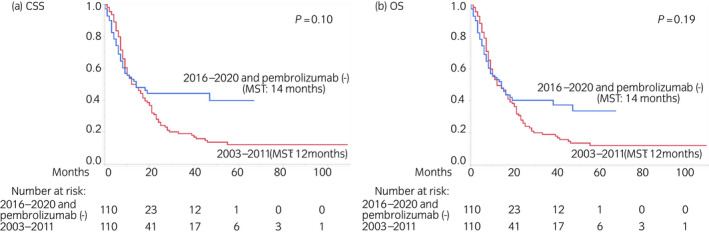
Kaplan–Meier curves after PSM depicting (a) CSS and (b) OS according to the era and overall pembrolizumab use (2016–2020 without pembrolizumab use vs. 2003–2011) in the reference analysis (Aim 2). CSS, cancer‐specific survival; MST, median survival time; OS, overall survival; PSM, propensity score matching.

Table S5 presents the characteristics of patients included in the reference analysis (Aim 2) before and after PSM. PSM was conducted as described for the primary analysis, and 220 patients were selected. Excluding the first‐line regimens, overall pembrolizumab use, and follow‐up duration, all of which were not matched between the two cohorts, no variables significantly differed between the cohorts. Figure 4 presents the Kaplan–Meier curves after PSM depicting CSS and OS according to the era and overall pembrolizumab use (2016–2020 without pembrolizumab use vs. 2003–2011). In total, there were 53 and 92 cancer‐specific deaths and 57 and 93 overall deaths in the recent and earlier era cohorts, respectively. In contrast to the aforementioned results, patients in the recent era cohort who did not receive pembrolizumab had similar outcomes (CSS and OS: 14 months) to those in the earlier era cohort (CSS and OS: 12 months). On multivariate analysis after PSM, ECOG PS (≤1 vs. ≥2), resection of the primary site (no vs. yes), and liver metastasis (no vs. yes) were identified as independent prognostic factors for both CSS and OS, whereas the era (2003–2011 vs. 2016–2020) was not associated with outcomes in univariate or multivariate analysis (Table S6).

## DISCUSSION

In the present study, we compared the survival outcomes of real‐world patients with aUC treated with salvage chemotherapy before and after the induction of pembrolizumab using propensity score‐matched multicenter cohorts for the primary analysis (Aim 1). Both before and after PSM, significant improvements in survival were demonstrated in the recent era (2016–2020) cohort, including an almost 2‐fold increase of CSS (from 12–13 months to 21–22 months, Figure 2; Figure S2). Furthermore, to estimate the direct survival‐prolonging effect of pembrolizumab, we also compared the outcomes of patients in the recent era cohort according to their receipt or non‐receipt of pembrolizumab with those of patients in the earlier era cohort as the secondary analysis (Aim 2). After PSM, patients in the recent era cohort who received pembrolizumab had significantly longer survival than those in the earlier era cohort, whereas patients in the recent era cohort who did not receive pembrolizumab had similar outcomes to those in the earlier era cohort, suggesting that pembrolizumab contributed to the improvements in survival. Notably, CSS (25 months) and OS (24 months) among patients in the recent era cohort who received pembrolizumab were more than 2‐fold longer than in patients in the earlier era cohort (CSS and OS: 11 months, Figure 3).

Since pembrolizumab was launched as a second‐line therapy for aUC in 2017, several studies have reported its outcomes in the real‐world setting,^14^, ^15^, ^16^, ^17^, ^18^, ^19^, ^20^ and all but one study conducted survival analyses from the start of pembrolizumab therapy.^15^, ^16^, ^17^, ^18^, ^19^, ^20^ The remaining study, by Narita et al., performed survival analyses from the start of first‐line chemotherapy to compare outcomes between pembrolizumab and conventional chemotherapy as later‐line therapies for aUC.^14^ After excluding patients who received first‐line chemotherapy alone (*n* = 69), the researchers compared OS between patients who received pembrolizumab (*n* = 121) and those who received chemotherapy alone (*n* = 67) as later‐line treatment. They reported median OS times of 24.7 and 16.3 months in the pembrolizumab and chemotherapy groups, respectively, and the difference was significant (*p* = 0.003) on multivariate Cox proportional hazards analysis using the inverse probability of treatment weighting method.^14^ Although our results are generally in line with these findings, their OS times were slightly longer than ours, which might be attributable to selection bias. Because the study by Narita et al. excluded patients who received first‐line chemotherapy alone, patients who did not receive second‐line therapy because of death following rapid progression after first‐line chemotherapy were not included in the analysis.^14^ Another study by Isobe et al. compared the outcomes of second‐line treatment for aUC provided before and after the approval of pembrolizumab, although its survival analyses were conducted from the start of pembrolizumab therapy.^20^ In this study, all 104 patients in the pre‐pembrolizumab group received gemcitabine/docetaxel as the second‐line therapy. Meanwhile, 79 of 94 (84%) patients in the post‐pembrolizumab group received pembrolizumab, and the remaining 15 (16%) patients received gemcitabine/docetaxel. The authors reported that OS was significantly longer in the post‐pembrolizumab group (13.6 months) than in the pre‐pembrolizumab group (7.6 months, *p* < 0.01).^20^ Although the start of the survival analyses differed, our results were consistent with those of the aforementioned study.

Concerning prognostic factors other than the era, ECOG PS ≥2 and liver metastasis were consistently identified as independent predictors of poor CSS and OS in all four analyses (Table 2; Tables [Link], [Link], and S6). These two factors have been recognized as strong prognostic markers for aUC, and their utility has been maintained in the pembrolizumab era (note: liver metastasis was previously included in visceral metastasis along with lung and bone metastases, but it has been handled separately in recent years).^1^, ^2^, ^3^, ^4^, ^5^, ^6^, ^7^, ^8^, ^9^, ^10^, ^11^, ^14^, ^15^, ^16^, ^17^, ^18^, ^19^, ^20^ Conversely, resection of the primary site was associated with survival in analyses of the entire cohort (Table 2; Table S2) and a subgroup analysis of patients who did not receive pembrolizumab (Table S6), whereas it had no correlation with survival in a subgroup analysis of patients who used pembrolizumab (Table S4). This might be attributable to the functional mechanism of immune checkpoint inhibitors such as pembrolizumab, the responders to which could benefit irrespective of the residual tumor burden, thus negating the cytoreductive effect of resection of the primary site. In fact, a previous study conducted before pembrolizumab approval reported a possible correlation of resection of the primary site with survival,^9^ whereas no recent studies assessing the outcomes of pembrolizumab identified an association with survival outcomes.^15^, ^17^, ^19^

The limitations of this study included its retrospective design and relatively short follow‐up period especially in the recent era cohort. Furthermore, because of the multicenter nature of this study, the characteristics and treatment patterns of patients were heterogeneous and detailed information on the following respects was missing: (1) reasons for inexecution of pembrolizumab in patients who did not undergo pembrolizumab in the recent era cohort; (2) duration of pembrolizumab therapy in patients who underwent pembrolizumab in the recent era cohort; and (3) subsequent therapy after pembrolizumab (for reference, since follow‐up information was obtained as of November 2021 for the recent era cohort, no patient in the present study received enfortumab vedotin which started to be sold on November 30, 2021 in Japan). Based on the total number of patients (*n* = 531), this was a medium‐sized study, but it is one of the largest cohorts in the field of aUC.^17^ Although this study demonstrated improved survival in aUC, its prognosis remains dismal, and thus, further improvements involving the development of novel therapeutic agents (e.g. avelumab and enfortumab vedotin) and/or optimization of the treatment strategy are needed.

In conclusion, both before and after PSM, patients with aUC treated in the recent era (2016–2020) displayed significantly longer CSS and OS than those treated before the drug became available (2003–2011). The improvement in survival could mainly be attributed to the survival‐prolonging effect of pembrolizumab.

## AUTHOR CONTRIBUTIONS

S.T. and T.K. contributed to the conception, study design, analysis, interpretation of data, and drafted the first manuscript. T.N. contributed to the conception, study design, supervision, and revised the manuscript critically for important intellectual content. J.M., K.K., K.S., Y.N., J.K., D.O., K.Y., T.K., K.Y., S.Y., S.K., K.K., Y.S., M.T., A.M., Y.A., Y.Y., Y.S., and D.Y. contributed to acquisition of data. Y.U. contributed to the analysis and interpretation of data. Y.E., H.N., A.I., Y.T., Y.N., T.F., H.F., S.T., and H.K. supervised the study, helped to draft the manuscript and were involved in revising it critically for important intellectual content. All authors read and approved the final manuscript.

## CONFLICT OF INTEREST

None declared

## APPROVAL OF THE RESEARCH PROTOCOL BY AN INSTITUTIONAL REVIEWER BOARD

This study was approved by the Institutional Review Board of the Graduate School of Medicine and Faculty of Medicine, The University of Tokyo (approval number: 10565), as well as that of each participating institution.

## INFORMED CONSENT

Because of the retrospective design of the study, the need for written informed consent was waived.

## REGISTRY AND THE REGISTRATION NO. OF THE STUDY/TRIAL

N/A.

## ANIMAL STUDIES

N/A.

## Supporting information


Figure S1.

Figure S2.

**Table S1.** Detailed patient demographics by institution (*n* = 531)

**Table S2.** Univariate and multivariate Cox proportional hazard regression analyses of CSS and OS before PSM (*n* = 531)

**Table S3.** Characteristics of patients in the secondary analysis (Aim 2) before and after PSM

**Table S4.** Univariate and multivariate Cox proportional hazard regression analyses of CSS and OS in the secondary analysis (Aim 2; *n* = 258)

**Table S5.** Characteristics of patients in the reference analysis (Aim 2) before and after PSM

**Table S6.** Univariate and multivariate Cox proportional hazard regression analyses of CSS and OS in the reference analysis (Aim 2; *n* = 220)
