# Supplementary figures and images for: Improved survival in real‐world patients with advanced urothelial carcinoma: A multicenter propensity score‐matched cohort study comparing a period before the introduction of pembrolizumab (2003–2011) and a more recent period (2016–2020)
Source: Int J Urol. 2022 Aug 22;29(12):1462–9. doi: 10.1111/iju.15014 (PMC10087413; doi:10.1111/iju.15014)

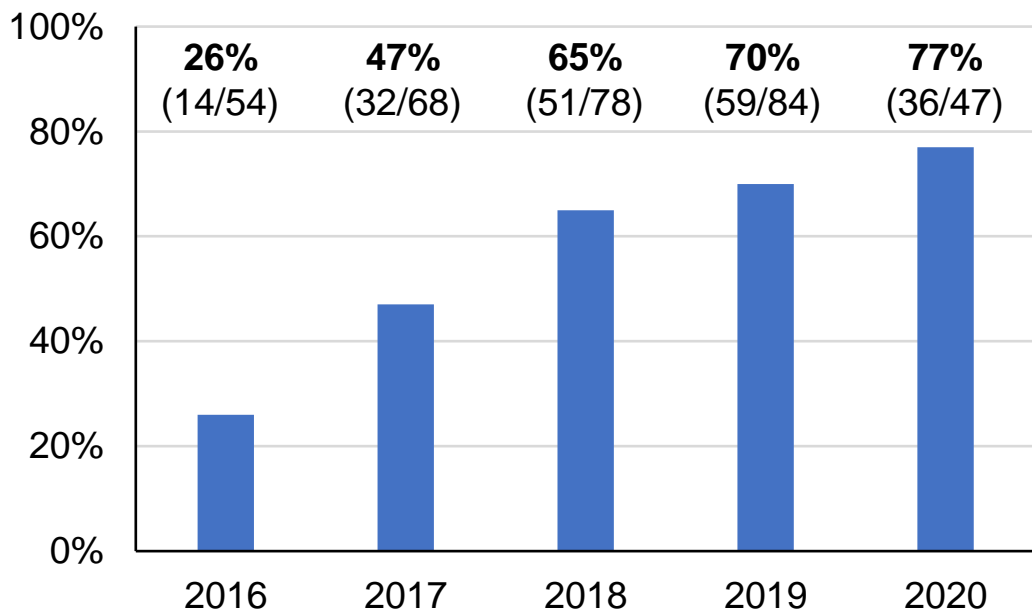

Supplement: Supplementary file 1 — Figure S1. [file IJU-29-1462-s008.pdf]
