# Supplementary material for: Improved survival in real‐world patients with advanced urothelial carcinoma: A multicenter propensity score‐matched cohort study comparing a period before the introduction of pembrolizumab (2003–2011) and a more recent period (2016–2020)
Source: Int J Urol. 2022 Aug 22;29(12):1462–9. doi: 10.1111/iju.15014 (PMC10087413; doi:10.1111/iju.15014)
Supplement: Supplementary file 2 — Figure S2. [file IJU-29-1462-s004.pdf]

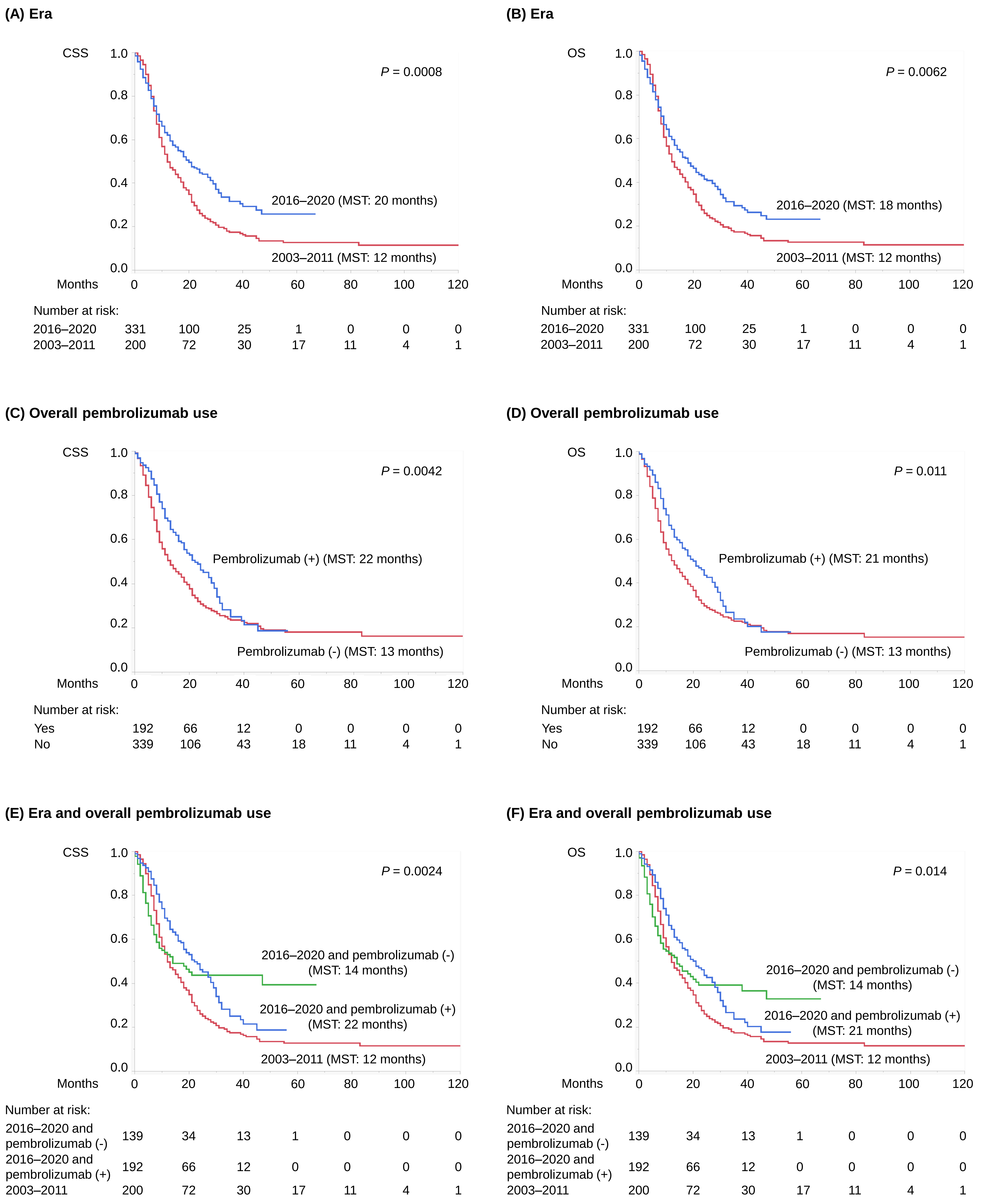
(A) Era
CSS
1.0
0.8
0.6
0.4
0.2
0.0
P = 0.0008
2016–2020 (MST: 20 months)
2003–2011 (MST: 12 months)
Months 0 20 40 60 80 100 120
Number at risk:
2016–2020 331 100 25 1 0 0 0
2003–2011 200 72 30 17 11 4 1
(B) Era
OS
1.0
0.8
0.6
0.4
0.2
0.0
P = 0.0062
2016–2020 (MST: 18 months)
2003–2011 (MST: 12 months)
Months 0 20 40 60 80 100 120
Number at risk:
2016–2020 331 100 25 1 0 0 0
2003–2011 200 72 30 17 11 4 1
(C) Overall pembrolizumab use
CSS
1.0
0.8
0.6
0.4
0.2
0.0
P = 0.0042
Pembrolizumab (+) (MST: 22 months)
Pembrolizumab (-) (MST: 13 months)
Months 0 20 40 60 80 100 120
Number at risk:
Yes 192 66 12 0 0 0 0
No 339 106 43 18 11 4 1
(D) Overall pembrolizumab use
OS
1.0
0.8
0.6
0.4
0.2
0.0
P = 0.011
Pembrolizumab (+) (MST: 21 months)
Pembrolizumab (-) (MST: 13 months)
Months 0 20 40 60 80 100 120
Number at risk:
Yes 192 66 12 0 0 0 0
No 339 106 43 18 11 4 1
(E) Era and overall pembrolizumab use
CSS
1.0
0.8
0.6
0.4
0.2
0.0
P = 0.0024
2016–2020 and pembrolizumab (-)
(MST: 14 months)
2016–2020 and pembrolizumab (+)
(MST: 22 months)
2003–2011 (MST: 12 months)
Months 0 20 40 60 80 100 120
Number at risk:
2016–2020 and pembrolizumab (-) 139 34 13 1 0 0 0
2016–2020 and pembrolizumab (+) 192 66 12 0 0 0 0
2003–2011 200 72 30 17 11 4 1
(F) Era and overall pembrolizumab use
OS
1.0
0.8
0.6
0.4
0.2
0.0
P = 0.014
2016–2020 and pembrolizumab (-)
(MST: 14 months)
2016–2020 and pembrolizumab (+)
(MST: 21 months)
2003–2011 (MST: 12 months)
Months 0 20 40 60 80 100 120
Number at risk:
2016–2020 and pembrolizumab (-) 139 34 13 1 0 0 0
2016–2020 and pembrolizumab (+) 192 66 12 0 0 0 0
2003–2011 200 72 30 17 11 4 1
