# Supplementary material for: Improved survival in real‐world patients with advanced urothelial carcinoma: A multicenter propensity score‐matched cohort study comparing a period before the introduction of pembrolizumab (2003–2011) and a more recent period (2016–2020)
Source: Int J Urol. 2022 Aug 22;29(12):1462–9. doi: 10.1111/iju.15014 (PMC10087413; doi:10.1111/iju.15014)
Supplement: Supplementary file 3 — Table S1. Detailed patient demographics by institution (n = 531) [file IJU-29-1462-s005.docx]

**Table S1** Detailed patient demographics by institution (*n* = 531)

| Institution | Total (*n*=531) | 2016–2020 (*n*=331) | 2003–2011 (*n*=200) |
| --- | --- | --- | --- |
| The University of Tokyo | 153 | 70 | 83 |
| Kyorin University School of Medicine | 91 | 91 | – |
| Jichi Medical University | 52 | 52 | – |
| Tokyo Metropolitan Tama Medical Center | 48 | – | 48 |
| Mitsui Memorial Hospital | 47 | 28 | 19 |
| Nihon University School of Medicine | 38 | 38 | – |
| The Fraternity Memorial Hospital | 30 | 30 | – |
| Musashino Red Cross Hospital | 27 | – | 27 |
| Tokyo Teishin Hospital | 23 | – | 23 |
| Teikyo University School of Medicine | 22 | 22 | – |
