# Supplementary material for: Improved survival in real‐world patients with advanced urothelial carcinoma: A multicenter propensity score‐matched cohort study comparing a period before the introduction of pembrolizumab (2003–2011) and a more recent period (2016–2020)
Source: Int J Urol. 2022 Aug 22;29(12):1462–9. doi: 10.1111/iju.15014 (PMC10087413; doi:10.1111/iju.15014)
Supplement: Supplementary file 4 — Table S2. Univariate and multivariate Cox proportional hazard regression analyses of CSS and OS before PSM (n = 531) [file IJU-29-1462-s002.docx]

**Table S2** Univariate and multivariate Cox proportional hazard regression analyses of CSS and OS before PSM (*n* = 531)

| Parameter | Cutoff | CSS Univariate | | CSS Multivariate | | OS Univariate | | OS Multivariate | |
| --- | --- | --- | --- | --- | --- | --- | --- | --- | --- |
|  |  | HR (95% CI) | *P* | HR (95% CI) | *P* | HR (95% CI) | *P* | HR (95% CI) | *P* |
| Age (years) | Continuous | 1.01 (1.00 to 1.02) per score | 0.042^*^ | 1.01 (1.00 to 1.03) per score | 0.039^*^ | 1.01 (1.00 to 1.03) per score | 0.0147^*^ | 1.02 (1.00 to 1.03) per score | 0.017^*^ |
| Sex | Male | Reference | 0.87 |  |  | Reference | 0.92 |  |  |
|  | Female | 1.02 (0.79 to 1.33) |  |  |  | 0.99 (0.76 to 1.28) |  |  |  |
| ECOG PS | ≤1 | Reference | < 0.0001^*^ | Reference | < 0.0001^*^ | Reference | < 0.0001^*^ | Reference | < 0.0001^*^ |
|  | ≥2 | 3.45 (2.47 to 4.82) |  | 2.72 (1.90 to 3.89) |  | 3.46 (2.50 to 4.80) |  | 2.74 (1.93 to 3.89) |  |
| Primary site | Bladder | Reference | 0.89 |  |  | Reference | 0.95 |  |  |
|  | Upper urinary tract | 1.06 (0.84 to 1.33) |  |  |  | 1.02 (0.82 to 1.28) |  |  |  |
|  | Both | 1.03 (0.73 to 1.46) |  |  |  | 1.05 (0.75 to 1.47) |  |  |  |
| Resection of primary site | No | Reference | 0.0015^*^ | Reference | 0.0046^*^ | Reference | 0.0016^*^ | Reference | 0.0052^*^ |
|  | Yes | 0.70 (0.56 to 0.87) |  | 0.73 (0.58 to 0.91) |  | 0.71 (0.57 to 0.88) |  | 0.73 (0.59 to 0.91) |  |
| Prior neoadjuvant/adjuvant chemotherapy | No | Reference | 0.74 |  |  | Reference | 0.61 |  |  |
|  | Yes | 0.96 (0.76 to 1.21) |  |  |  | 0.94 (0.75 to 1.18) |  |  |  |
| Lymph node metastasis | No | Reference | 0.29 |  |  | Reference | 0.41 |  |  |
|  | Yes | 1.13 (0.90 to 1.42) |  |  |  | 1.10 (0.88 to 1.37) |  |  |  |
| Lung metastasis | No | Reference | 0.18 |  |  | Reference | 0.18 |  |  |
|  | Yes | 1.16 (0.93 to 1.45) |  |  |  | 1.16 (0.93 to 1.44) |  |  |  |
| Bone metastasis | No | Reference | 0.0003^*^ | Reference | 0.096 | Reference | 0.0003^*^ | Reference | 0.11 |
|  | Yes | 1.67 (1.27 to 2.20) |  | 1.28 (0.96 to 1.72) |  | 1.65 (1.25 to 2.16) |  | 1.27 (0.95 to 1.69) |  |
| Liver metastasis | No | Reference | < 0.0001^*^ | Reference | < 0.0001^*^ | Reference | < 0.0001^*^ | Reference | < 0.0001^*^ |
|  | Yes | 2.97 (2.20 to 4.01) |  | 2.53 (1.85 to 3.46) |  | 2.88 (2.14 to 3.87) |  | 2.44 (1.79 to 3.32) |  |
| First-line regimens | GC | Reference | 0.84 |  |  | Reference | 0.85 |  |  |
|  | GCa | 1.00 (0.73 to 1.38) |  |  |  | 0.96 (0.70 to 1.32) |  |  |  |
|  | MVAC | 1.15 (0.85 to 1.55) |  |  |  | 1.10 (0.82 to 1.48) |  |  |  |
|  | ddMVAC | 1.09 (0.48 to 2.48) |  |  |  | 1.03 (0.46 to 2.33) |  |  |  |
|  | Pembrolizumab | 1.15 (0.77 to 1.71) |  |  |  | 1.15 (0.78 to 1.70) |  |  |  |
|  | Others | 1.21 (0.87 to 1.70) |  |  |  | 1.20 (0.86 to 1.66) |  |  |  |
| Era | 2003–2011 | Reference | 0.0011^*^ | Reference | 0.011^*^ | Reference | 0.0076^*^ | Reference | 0.032^*^ |
|  | 2016–2020 | 0.70 (0.57 to 0.87) |  | 0.68 (0.51 to 0.92) |  | 0.75 (0.61 to 0.93) |  | 0.73 (0.55 to 0.97) |  |
| Overall pembrolizumab use | No | Reference | 0.0052^*^ | Reference | 0.27 | Reference | 0.013^*^ | Reference | 0.24 |
|  | Yes | 0.72 (0.57 to 0.91) |  | 0.84 (0.61 to 1.14) |  | 0.75 (0.60 to 0.94) |  | 0.84 (0.62 to 1.13) |  |

CI, confidence interval; CSS, cancer-specific survival; ddMVAC, dose-dense methotrexate/vinblastine/doxorubicin/cisplatin; ECOG PS, Eastern Cooperative Oncology Group performance status; GC, gemcitabine/cisplatin; GCa, gemcitabine/carboplatin; HR, hazard ratio; IQR, interquartile range; MVAC, methotrexate/vinblastine/doxorubicin/cisplatin; OS, overall survival; PSM, propensity score matching; ^*^, statistically significant
