# Supplementary material for: Improved survival in real‐world patients with advanced urothelial carcinoma: A multicenter propensity score‐matched cohort study comparing a period before the introduction of pembrolizumab (2003–2011) and a more recent period (2016–2020)
Source: Int J Urol. 2022 Aug 22;29(12):1462–9. doi: 10.1111/iju.15014 (PMC10087413; doi:10.1111/iju.15014)
Supplement: Supplementary file 5 — Table S3. Characteristics of patients in the secondary analysis (Aim 2) before and after PSM [file IJU-29-1462-s006.docx]

**Table S3** Characteristics of patients in the secondary analysis (Aim 2) before and after PSM

|  | Before PSM | | | | After PSM | | | |
| --- | --- | --- | --- | --- | --- | --- | --- | --- |
| Parameter | Total (*n*=392) | 2016–2020 and pembrolizumab (+) (*n*=192) | 2003–2011 (*n*=200) | *P*-value | Total (*n*=258) | 2016–2020 and pembrolizumab (+) (*n*=129) | 2003–2011 (*n*=129) | *P*-value |
| Age, years, median (IQR) | 70 (63–75) | 71 (66–76) | 68 (62–74) | 0.0086^a*^ | 70 (65–76) | 70 (65–76) | 70 (64–75) | 0.87^a^ |
| Sex, no. (%) |  |  |  | 0.56^b^ |  |  |  | 0.87^b^ |
| Male | 309 (78.8) | 149 (77.6) | 160 (80.0) |  | 209 (81.0) | 104 (80.6) | 105 (81.4) |  |
| Female | 83 (21.2) | 43 (22.4) | 40 (20.0) |  | 49 (19.0) | 25 (19.4) | 24 (18.6) |  |
| ECOG PS, no. (%) |  |  |  | 0.62^b^ |  |  |  | 0.49^b^ |
| ≤1 | 360 (91.8) | 175 (91.2) | 185 (92.5) |  | 237 (91.9) | 120 (93.0) | 117 (90.7) |  |
| ≥2 | 32 (8.2) | 17 (8.9) | 15 (7.5) |  | 21 (8.1) | 9 (7.0) | 12 (9.3) |  |
| Primary site, no. (%) |  |  |  | 0.30^b^ |  |  |  | 0.75^b^ |
| Bladder | 179 (45.7) | 82 (42.7) | 97 (48.5) |  | 121 (46.9) | 58 (45.0) | 63 (48.8) |  |
| Upper urinary tract | 163 (41.6) | 81 (42.2) | 82 (41.0) |  | 104 (40.3) | 55 (42.6) | 49 (38.0) |  |
| Both | 50 (12.8) | 29 (15.1) | 21 (10.5) |  | 33 (12.8) | 16 (12.4) | 17 (13.2) |  |
| Resection of primary site, no. (%) | 264 (67.4) | 127 (66.2) | 137 (68.5) | 0.62^b^ | 168 (65.1) | 82 (63.6) | 86 (66.7) | 0.60^b^ |
| Prior neoadjuvant/adjuvant chemotherapy, no. (%) | 137 (35.0) | 85 (44.3) | 52 (26.0) | 0.0001^b*^ | 82 (31.8) | 39 (30.2) | 43 (33.3) | 0.59^b^ |
| Lymph node metastasis, no. (%) | 253 (64.5) | 116 (60.4) | 137 (68.5) | 0.095^b^ | 165 (64.0) | 84 (65.1) | 81 (62.8) | 0.70^b^ |
| Visceral metastasis, no. (%) | 219 (55.9) | 115 (59.9) | 104 (52.0) | 0.12^b^ | 145 (56.2) | 70 (54.3) | 75 (58.1) | 0.53^b^ |
| Lung metastasis, no. (%) | 135 (34.4) | 64 (33.3) | 71 (35.5) | 0.65^b^ | 90 (34.9) | 43 (33.3) | 47 (36.4) | 0.60^b^ |
| Bone metastasis, no. (%) | 58 (14.8) | 27 (14.1) | 31 (15.5) | 0.69^b^ | 38 (14.7) | 16 (12.4) | 22 (17.1) | 0.29^b^ |
| Liver metastasis, no. (%) | 45 (11.5) | 22 (11.5) | 23 (11.5) | 0.99^b^ | 31 (12.0) | 14 (10.9) | 17 (13.2) | 0.57^b^ |
| First-line regimens, no. (%) |  |  |  | < 0.0001^b*^ |  |  |  | < 0.0001^b*^ |
| GC | 160 (40.8) | 68 (35.4) | 92 (46.0) |  | 114 (44.2) | 55 (42.6) | 59 (45.7) |  |
| GCa | 50 (12.8) | 50 (26.0) | 0 (0) |  | 32 (12.4) | 32 (24.8) | 0 (0) |  |
| MVAC | 76 (19.4) | 4 (2.1) | 72 (36.0) |  | 50 (19.4) | 4 (3.1) | 46 (35.7) |  |
| ddMVAC | 5 (1.3) | 5 (2.6) | 0 (0) |  | 5 (1.9) | 5 (3.9) | 0 (0) |  |
| Pembrolizumab | 53 (13.5) | 53 (27.6) | 0 (0) |  | 26 (10.1) | 26 (20.2) | 0 (0) |  |
| Others | 48 (12.2) | 12 (6.3) | 36 (18.0) |  | 31 (12.0) | 7 (5.4) | 24 (18.6) |  |
| Overall pembrolizumab use, no. (%) | 192 (49.0) | 192 (100) | 0 (0) | < 0.0001^b*^ | 129 (50.0) | 129 (100) | 0 (0) | < 0.0001^b*^ |
| Follow-up duration, months, median (IQR) | 13 (7–24) | 15 (8–24) | 12 (7–25) | 0.015^a*^ | 13 (7–24) | 16 (8–25) | 11 (7–22) | 0.84^a^ |

ddMVAC, dose-dense methotrexate/vinblastine/doxorubicin/cisplatin; ECOG PS, Eastern Cooperative Oncology Group performance status; GC, gemcitabine/cisplatin; GCa, gemcitabine/carboplatin; IQR, interquartile range; MVAC, methotrexate/vinblastine/doxorubicin/cisplatin; PSM, propensity score matching; ^*^, statistically significant; ^a^, Student’s *t*-test; ^b^, χ^2^ test
