# Supplementary material for: Improved survival in real‐world patients with advanced urothelial carcinoma: A multicenter propensity score‐matched cohort study comparing a period before the introduction of pembrolizumab (2003–2011) and a more recent period (2016–2020)
Source: Int J Urol. 2022 Aug 22;29(12):1462–9. doi: 10.1111/iju.15014 (PMC10087413; doi:10.1111/iju.15014)
Supplement: Supplementary file 6 — Table S4. Univariate and multivariate Cox proportional hazard regression analyses of CSS and OS in the secondary analysis (Aim 2; n = 258) [file IJU-29-1462-s001.docx]

**Table S4** Univariate and multivariate Cox proportional hazard regression analyses of CSS and OS in the secondary analysis (Aim 2; *n* = 258)

| Parameter | Cutoff | CSS Univariate | | CSS Multivariate | | OS Univariate | | OS Multivariate | |
| --- | --- | --- | --- | --- | --- | --- | --- | --- | --- |
|  |  | HR (95% CI) | *P* | HR (95% CI) | *P* | HR (95% CI) | *P* | HR (95% CI) | *P* |
| Age (years) | Continuous | 1.02 (1.00 to 1.03) per score | 0.055 |  |  | 1.02 (1.00 to 1.03) per score | 0.048^*^ | 1.02 (1.00 to 1.04) per score | 0.068 |
| Sex | Male | Reference | 0.77 |  |  | Reference | 0.65 |  |  |
|  | Female | 0.95 (0.65 to 1.38) |  |  |  | 0.92 (0.63 to 1.33) |  |  |  |
| ECOG PS | ≤1 | Reference | < 0.0001^*^ | Reference | 0.0002^*^ | Reference | < 0.0001^*^ | Reference | 0.0002^*^ |
|  | ≥2 | 3.69 (2.27 to 6.00) |  | 2.77 (1.63 to 4.70) |  | 3.56 (2.19 to 5.78) |  | 2.73 (1.61 to 4.61) |  |
| Primary site | Bladder | Reference | 0.67 |  |  | Reference | 0.76 |  |  |
|  | Upper urinary tract | 1.06 (0.77 to 1.44) |  |  |  | 1.05 (0.77 to 1.42) |  |  |  |
|  | Both | 1.23 (0.78 to 1.94) |  |  |  | 1.19 (0.75 to 1.86) |  |  |  |
| Resection of primary site | No | Reference | 0.11 |  |  | Reference | 0.070 |  |  |
|  | Yes | 0.78 (0.58 to 1.05) |  |  |  | 0.76 (0.57 to 1.02) |  |  |  |
| Prior neoadjuvant/adjuvant chemotherapy | No | Reference | 0.68 |  |  | Reference | 0.61 |  |  |
|  | Yes | 0.94 (0.69 to 1.28) |  |  |  | 0.92 (0.68 to 1.25) |  |  |  |
| Lymph node metastasis | No | Reference | 0.11 |  |  | Reference | 0.089 |  |  |
|  | Yes | 1.28 (0.95 to 1.74) |  |  |  | 1.30 (0.96 to 1.76) |  |  |  |
| Lung metastasis | No | Reference | 0.72 |  |  | Reference | 0.76 |  |  |
|  | Yes | 0.94 (0.70 to 1.28) |  |  |  | 0.95 (0.71 to 1.29) |  |  |  |
| Bone metastasis | No | Reference | 0.061 |  |  | Reference | 0.089 |  |  |
|  | Yes | 1.44 (0.98 to 2.11) |  |  |  | 1.39 (0.95 to 2.04) |  |  |  |
| Liver metastasis | No | Reference | < 0.0001^*^ | Reference | 0.0004^*^ | Reference | < 0.0001^*^ | Reference | 0.0002^*^ |
|  | Yes | 2.72 (1.80 to 4.13) |  | 2.28 (1.45 to 3.60) |  | 2.74 (1.82 to 4.13) |  | 2.33 (1.49 to 3.63) |  |
| First-line regimens | GC | Reference | 0.60 |  |  | Reference | 0.58 |  |  |
|  | GCa | 0.66 (0.39 to 1.13) |  |  |  | 0.64 (0.38 to 1.09) |  |  |  |
|  | MVAC | 1.10 (0.76 to 1.59) |  |  |  | 1.09 (0.76 to 1.57) |  |  |  |
|  | ddMVAC | 0.93 (0.29 to 2.96) |  |  |  | 0.90 (0.28 to 2.84) |  |  |  |
|  | Pembrolizumab | 0.88 (0.48 to 1.61) |  |  |  | 0.91 (0.51 to 1.63) |  |  |  |
|  | Others | 1.13 (0.73 to 1.74) |  |  |  | 1.09 (0.71 to 1.67) |  |  |  |
| Era / Overall pembrolizumab use | 2003–2011 / No | Reference | < 0.0001^*^ | Reference | < 0.0001^*^ | Reference | < 0.0001^*^ | Reference | < 0.0001^*^ |
|  | 2016–2020 / Yes | 0.53 (0.39 to 0.71) |  | 0.50 (0.37 to 0.68) |  | 0.55 (0.41 to 0.74) |  | 0.53 (0.40 to 0.72) |  |

CI, confidence interval; CSS, cancer-specific survival; ddMVAC, dose-dense methotrexate/vinblastine/doxorubicin/cisplatin; ECOG PS, Eastern Cooperative Oncology Group performance status; GC, gemcitabine/cisplatin; GCa, gemcitabine/carboplatin; HR, hazard ratio; IQR, interquartile range; MVAC, methotrexate/vinblastine/doxorubicin/cisplatin; OS, overall survival; ^*^, statistically significant
