# Supplementary material for: Improved survival in real‐world patients with advanced urothelial carcinoma: A multicenter propensity score‐matched cohort study comparing a period before the introduction of pembrolizumab (2003–2011) and a more recent period (2016–2020)
Source: Int J Urol. 2022 Aug 22;29(12):1462–9. doi: 10.1111/iju.15014 (PMC10087413; doi:10.1111/iju.15014)
Supplement: Supplementary file 7 — Table S5. Characteristics of patients in the reference analysis (Aim 2) before and after PSM [file IJU-29-1462-s003.docx]

**Table S5** Characteristics of patients in the reference analysis (Aim 2) before and after PSM

|  | Before PSM | | | | After PSM | | | |
| --- | --- | --- | --- | --- | --- | --- | --- | --- |
| Parameter | Total (*n*=339) | 2016–2020 and pembrolizumab (-) (*n*=139) | 2003–2011 (*n*=200) | *P*-value | Total (*n*=220) | 2016–2020 and pembrolizumab (-) (*n*=110) | 2003–2011 (*n*=110) | *P*-value |
| Age, years, median (IQR) | 71 (64–76) | 73 (69–79) | 68 (62–74) | < 0.0001^a*^ | 73 (67–76) | 73 (67–76) | 73 (67–77) | 0.78^a^ |
| Sex, no. (%) |  |  |  | 0.41^b^ |  |  |  | 0.74^b^ |
| Male | 266 (78.5) | 106 (76.3) | 160 (80.0) |  | 172 (78.2) | 85 (77.3) | 87 (79.1) |  |
| Female | 73 (21.5) | 33 (23.7) | 40 (20.0) |  | 48 (21.8) | 25 (22.7) | 23 (20.9) |  |
| ECOG PS, no. (%) |  |  |  | 0.040^b*^ |  |  |  | 1.00^b^ |
| ≤1 | 304 (89.7) | 119 (85.6) | 185 (92.5) |  | 196 (89.1) | 98 (89.1) | 98 (89.1) |  |
| ≥2 | 35 (10.3) | 20 (14.4) | 15 (7.5) |  | 24 (10.9) | 12 (10.9) | 12 (10.9) |  |
| Primary site, no. (%) |  |  |  | 0.38^b^ |  |  |  | 0.90^b^ |
| Bladder | 175 (51.6) | 78 (56.1) | 97 (48.5) |  | 111 (50.5) | 56 (50.9) | 55 (50.0) |  |
| Upper urinary tract | 131 (38.6) | 49 (35.3) | 82 (41.0) |  | 87 (39.6) | 44 (40.0) | 43 (39.1) |  |
| Both | 33 (9.7) | 12 (8.6) | 21 (10.5) |  | 22 (10.0) | 10 (9.1) | 12 (10.9) |  |
| Resection of primary site, no. (%) | 214 (63.1) | 77 (55.4) | 137 (68.5) | 0.014^b*^ | 126 (57.3) | 63 (57.3) | 63 (57.3) | 1.00^b^ |
| Prior neoadjuvant/adjuvant chemotherapy, no. (%) | 73 (21.5) | 21 (15.1) | 52 (26.0) | 0.016^b*^ | 39 (17.7) | 20 (18.2) | 19 (17.3) | 0.86^b^ |
| Lymph node metastasis, no. (%) | 232 (68.4) | 95 (68.4) | 137 (68.5) | 0.98^b^ | 147 (66.8) | 74 (67.3) | 73 (66.4) | 0.89^b^ |
| Visceral metastasis, no. (%) | 175 (51.6) | 71 (51.1) | 104 (52.0) | 0.87^b^ | 113 (51.4) | 57 (51.8) | 56 (50.9) | 0.89^b^ |
| Lung metastasis, no. (%) | 111 (32.7) | 40 (28.8) | 71 (35.5) | 0.19^b^ | 70 (31.8) | 35 (31.8) | 35 (31.8) | 1.00^b^ |
| Bone metastasis, no. (%) | 54 (15.9) | 23 (16.6) | 31 (15.5) | 0.80^b^ | 34 (15.5) | 18 (16.4) | 16 (14.6) | 0.71^b^ |
| Liver metastasis, no. (%) | 36 (10.6) | 13 (9.4) | 23 (11.5) | 0.53^b^ | 23 (10.5) | 13 (11.8) | 10 (9.1) | 0.51^b^ |
| First-line regimens, no. (%) |  |  |  | < 0.0001^b*^ |  |  |  | < 0.0001^b*^ |
| GC | 169 (49.9) | 77 (55.4) | 92 (46.0) |  | 111 (50.5) | 60 (54.6) | 51 (46.4) |  |
| GCa | 45 (13.3) | 45 (32.4) | 0 (0) |  | 36 (16.4) | 36 (32.7) | 0 (0) |  |
| MVAC | 73 (21.5) | 1 (0.7) | 72 (36.0) |  | 37 (16.8) | 1 (0.9) | 36 (32.7) |  |
| ddMVAC | 5 (1.5) | 5 (3.6) | 0 (0) |  | 3 (1.4) | 3 (2.7) | 0 (0) |  |
| Others | 47 (13.9) | 11 (7.9) | 36 (18.0) |  | 33 (15.0) | 10 (9.1) | 23 (20.9) |  |
| Follow-up duration, months, median (IQR) | 10 (5–22) | 8 (3–19) | 12 (7–25) | 0.0008^a*^ | 10 (5–22) | 8 (4–17) | 12 (7–24) | 0.016^a*^ |

ddMVAC, dose-dense methotrexate/vinblastine/doxorubicin/cisplatin; ECOG PS, Eastern Cooperative Oncology Group performance status; GC, gemcitabine/cisplatin; GCa, gemcitabine/carboplatin; IQR, interquartile range; MVAC, methotrexate/vinblastine/doxorubicin/cisplatin; PSM, propensity score matching; ^*^, statistically significant; ^a^, Student’s *t*-test; ^b^, χ^2^ test
