# Supplementary material for: Improved survival in real‐world patients with advanced urothelial carcinoma: A multicenter propensity score‐matched cohort study comparing a period before the introduction of pembrolizumab (2003–2011) and a more recent period (2016–2020)
Source: Int J Urol. 2022 Aug 22;29(12):1462–9. doi: 10.1111/iju.15014 (PMC10087413; doi:10.1111/iju.15014)
Supplement: Supplementary file 8 — Table S6. Univariate and multivariate Cox proportional hazard regression analyses of CSS and OS in the reference analysis (Aim 2; n = 220) [file IJU-29-1462-s007.docx]

**Table S6** Univariate and multivariate Cox proportional hazard regression analyses of CSS and OS in the reference analysis (Aim 2; *n* = 220)

| Parameter | Cutoff | CSS Univariate | | CSS Multivariate | | OS Univariate | | OS Multivariate | |
| --- | --- | --- | --- | --- | --- | --- | --- | --- | --- |
|  |  | HR (95% CI) | *P* | HR (95% CI) | *P* | HR (95% CI) | *P* | HR (95% CI) | *P* |
| Age (years) | Continuous | 1.01 (0.99 to 1.03) per score | 0.47 |  |  | 1.01 (0.99 to 1.03) per score | 0.30 |  |  |
| Sex | Male | Reference | 0.90 |  |  | Reference | 0.95 |  |  |
|  | Female | 1.03 (0.69 to 1.53) |  |  |  | 0.99 (0.66 to 1.47) |  |  |  |
| ECOG PS | ≤1 | Reference | < 0.0001^*^ | Reference | < 0.0001^*^ | Reference | < 0.0001^*^ | Reference | < 0.0001^*^ |
|  | ≥2 | 5.42 (3.29 to 8.94) |  | 3.43 (1.93 to 6.09) |  | 5.32 (3.23 to 8.76) |  | 3.45 (1.95 to 6.10) |  |
| Primary site | Bladder | Reference | 0.80 |  |  | Reference | 0.55 |  |  |
|  | Upper urinary tract | 0.97 (0.69 to 1.38) |  |  |  | 0.94 (0.66 to 1.32) |  |  |  |
|  | Both | 1.17 (0.69 to 1.98) |  |  |  | 1.25 (0.76 to 2.07) |  |  |  |
| Resection of primary site | No | Reference | 0.0008^*^ | Reference | 0.0039^*^ | Reference | 0.0013^*^ | Reference | 0.0057^*^ |
|  | Yes | 0.57 (0.41 to 0.79) |  | 0.61 (0.44 to 0.85) |  | 0.59 (0.43 to 0.81) |  | 0.63 (0.45 to 0.87) |  |
| Prior neoadjuvant/adjuvant chemotherapy | No | Reference | 0.39 |  |  | Reference | 0.29 |  |  |
|  | Yes | 0.83 (0.54 to 1.27) |  |  |  | 0.79 (0.52 to 1.22) |  |  |  |
| Lymph node metastasis | No | Reference | 0.32 |  |  | Reference | 0.27 |  |  |
|  | Yes | 1.20 (0.84 to 1.71) |  |  |  | 1.22 (0.86 to 1.73) |  |  |  |
| Lung metastasis | No | Reference | 0.44 |  |  | Reference | 0.41 |  |  |
|  | Yes | 1.15 (0.81 to 1.62) |  |  |  | 1.15 (0.82 to 1.62) |  |  |  |
| Bone metastasis | No | Reference | 0.015^*^ | Reference | 0.40 | Reference | 0.022^*^ | Reference | 0.46 |
|  | Yes | 1.72 (1.11 to 2.65) |  | 1.22 (0.77 to 1.95) |  | 1.66 (1.08 to 2.56) |  | 1.19 (0.75 to 1.89) |  |
| Liver metastasis | No | Reference | < 0.0001^*^ | Reference | 0.0044^*^ | Reference | < 0.0001^*^ | Reference | 0.0052^*^ |
|  | Yes | 3.15 (1.97 to 5.03) |  | 2.17 (1.27 to 3.68) |  | 3.06 (1.92 to 4.88) |  | 2.12 (1.25 to 3.60) |  |
| First-line regimens | GC | Reference | 0.49 |  |  | Reference | 0.57 |  |  |
|  | GCa | 1.27 (0.75 to 2.14) |  |  |  | 1.22 (0.72 to 2.04) |  |  |  |
|  | MVAC | 1.45 (0.95 to 2.22) |  |  |  | 1.41 (0.93 to 2.14) |  |  |  |
|  | ddMVAC | 1.43 (0.35 to 5.84) |  |  |  | 1.36 (0.33 to 5.54) |  |  |  |
|  | Others | 1.24 (0.78 to 1.97) |  |  |  | 1.17 (0.74 to 1.86) |  |  |  |
| Era | 2003–2011 | Reference | 0.11 |  |  | Reference | 0.21 |  |  |
|  | 2016–2020 | 0.76 (0.54 to 1.06) |  |  |  | 0.81 (0.58 to 1.12) |  |  |  |

CI, confidence interval; CSS, cancer-specific survival; ddMVAC, dose-dense methotrexate/vinblastine/doxorubicin/cisplatin; ECOG PS, Eastern Cooperative Oncology Group performance status; GC, gemcitabine/cisplatin; GCa, gemcitabine/carboplatin; HR, hazard ratio; IQR, interquartile range; MVAC, methotrexate/vinblastine/doxorubicin/cisplatin; OS, overall survival; ^*^, statistically significant
